# A study of the provision of hospital based dental general anaesthetic services for children in the northwest of England: part 1 - a comparison of service delivery between six hospitals

**DOI:** 10.1186/s12903-015-0028-4

**Published:** 2015-04-18

**Authors:** Michaela Goodwin, Caroline Sanders, Iain A Pretty

**Affiliations:** The Dental Health Unit School of Dentistry, The University of Manchester, Williams House, Manchester Science Park, Manchester, M15 6SE UK; Centre for Primary Care The University of Manchester, Williamson Building, Oxford Road, Manchester, M13 9PL UK

**Keywords:** Caries, Children, Pain, Impact, General, Anaesthetic

## Abstract

**Background:**

Extensive caries in children can result in a referral for tooth extraction under General Anaesthesia (GA). While there are guidelines for the use of GA within paediatric dentistry this process is ultimately dependent upon the decision making of the treating dentist. This decision can be influenced locally by the availability of services and their waiting list. GA services for paediatric extractions (DGA) have developed from different historical positions, including community dental services, maxillofacial services and paediatric led specialist services.

**Methods:**

This article explores the differences between DGA services provided by 6 randomly selected hospitals across the North West of England. 456 patients who attended a routine DGA appointment in each hospital over a period of two months from 2012 to 2013 gave consent to allow access to their clinical notes and completed a questionnaire (93% consent rate). Data were entered onto SPSS and appropriate statistical tests undertaken.

**Results:**

Differences between hospitals included the clinic structure, patient characteristics and the treatment provided. There was a significant difference in the number of previous child DGAs experienced within the family, ranging from 33% to 59% across hospitals. Hospital 1 attendees differed in a number of ways to other areas but notably in the stability of life time residency with 20% of patients having previously lived in another area and with just 58% of parents stating their child regularly attended the dentist (compared to an average of 9% and 81% respectively across other hospitals).

**Conclusion:**

Findings suggest services throughout the region face different obstacles in providing support and treatment for young children referred for DGA. There are, however common practices such as preventative treatment, which could impact on caries experience and subsequent DGA referral, a particular issue given the high DGA repeat rate observed. For many children a DGA may be their first dental experience. It is therefore vital to engage with both child and family at this stage, attempt to initiate a pattern of dental attendance and to ensure this experience does not create an on-going cycle of poor dental behaviour and health.

**Electronic supplementary material:**

The online version of this article (doi:10.1186/s12903-015-0028-4) contains supplementary material, which is available to authorized users.

## Background

Despite the fall in caries prevalence over the last 40 years, strongly linked to the widespread use of fluoride toothpaste [1], tooth decay is still a significant problem. Dental decay is a global issue but has become concentrated in the most vulnerable section of society; namely young children and the most deprived [2]. Caries is a multi-factorial disease and its effects can range from mild discomfort, to on-going pain that affects quality of life [3]. If dental decay and subsequent infection or pain becomes too severe a dentist may elect to refer a child for tooth extraction under General Anaesthetic (GA). This should be reserved for the most severe cases, given the associated morbidity of the procedure, limits of service provision i.e. wait times within a hospital setting [4] and cost of the procedure estimated at £36,282,960 within the UK [5,6].

During the early 20th Century, extraction under general anaesthetic was a routine treatment option for managing decay in young children. Following a decrease in caries, levels of the number of dental general anaesthetics (DGA) in the UK also decreased [7]. In 2002, following a report entitled ‘A Conscious Decision’, general anaesthetics could only be provided within a secondary care setting. This was largely due to a number of fatalities following administration of GA within primary care settings [7,8]. The most recent report on hospital extractions by the Dental Health Observatory (now the Dental Public Health Intelligence Programme) produced data showing a rise in the number of extractions being carried out in a hospital setting throughout the North West of England, the majority of which under GA [9]. Additional data were released by the HSCIC (Heath and Social Care Information Centre), which showed a year on year increase of children attending hospital for caries treatment throughout England from 2010 to 2014 [10].

There are a variety of additional reasons, beyond the severity of dental caries, why children may be referred for a GA extraction, which may explain this rise against the context of falling disease levels. These range from the referring dentists’ skill and confidence in treating young children, to the services available in that area. One study, examining the experience and self assessed confidence of students at 3 dental schools, found confidence lowest for ‘selecting patients for GA’ [11]. Additionally, a number of studies have indicated there is an apparent lack of understanding from referring dentists as to the appropriate provision of DGA plus little adherence to the GDC guidelines surrounding this pathway [12]. The overall fall in disease on a population level, but with its concentration in the most deprived communities, appears not to have impacted on the level of DGA required i.e. prevalence has decreased but severe caries remains a significant public health issue.

Historically there is no clear defined pathway for young children with multiple decayed teeth although in recent years various care pathways have been suggested and guidelines for the use of GA in paediatric patients have been published [13,14]. It is recognised that due to differences in providers, commissioners and dental need/demand across geographic areas, a varied DGA service landscape and utilisation is seen [15].

As many of the services are provider led, and historical in nature, they may not have been commissioned based on a formal health needs assessment or have a clear service specification, unlike many contemporary services. Much of this reflects the legacy of these services, however, understanding the differences between them, and impact on children and families, is important. Such differences may also reveal elements of best practice that can form part of a service specification and commissioning.

### Aim

To explore differences in the DGA population and services provided for children admitted to six selected hospital sites for a dental extraction under GA in the North West of England and detail certain qualities that can be replicated across these services.

### Objectives

- To collect hospital data (wait times, etc.).
- To collect data gained from service users on their dental treatment (preventative treatment, previous GA) and certain features of this population related to oral health, delivering effective prevention and treatment (attendance, translator required).
- To observe the process of treatment under GA on the day of the operation.
- To observe any prevention or assessment that occurs before treatment under GA.

### Hypothesis

- There will be a significant difference between those attending different hospitals in key variables relating to both the population (IMD, language and attendance) and in treatment (number of teeth extracted and prevention advice given).
- There will be ‘best practice’ qualities of services, which should be replicated across other hospitals in relation to quality of care and the positive impact on children.

## Methods

Data were collected following recruitment of patients from six randomly selected hospitals (using a random number generator) across the North West. The criterion for a hospital to be included in the random sample was it had to carry out more than 200 operations for an extraction per year, 21 conformed to this criterion. Each hospital was visited for a period of two months on a series of rolling visits over a period of a year and a half during which the researcher (MG) attended every session scheduled for GA extraction, commonly known as ‘outpatient’ GA, i.e. those not limited to individuals with special treatment needs or requiring complex procedures. This permitted the research team to gain a representative sample from each hospital. The sample size was calculated from information gained both from the Dental Health Observatory (now the Dental Public Health Intelligence Programme) and from a previous service evaluation completed based on the ratio of male to female participants, an absolute precision of 5% and given a 95% Confidence. Other proportions with known data were calculated i.e. proportion of children seen who were 5 years and younger but as the ratio for male/female was almost 50:50 this yielded the largest, minimum sample size required.

$$ \frac{1{.96}^2\ (0.54)\ (0.46)14146}{0{.05}^2\ \left(14146 - 1\right) + 1{.96}^2\times 0.49\times 0.51} = 374\ \mathrm{participants} $$

Given the final sample size of 456 participants in this study there was deemed sufficient numbers for further analysis.

Data were gained from a variety of sources including a questionnaire completed by the parent which contained questions on dental history, sociodemographics and whether previous information or preventative treatment had been given. Additionally information was collected from clinical and referral notes which comprised of teeth requested and planned for extraction, recorded pain, anxiety, medical history, dates of referral as well as notes on any additional information recorded. Researcher notes were also made at each session regarding the process and delivery of the service these were framed around patient arrival and departure times, hospital setting/layout, staff available during the GA sessions and child friendly activities within the waiting area or equivalent. These were primarily recorded to assist with further questions during the qualitative interviews which explored experiences of DGA. Information gained during these note taking have been described in Additional file 1: Table S1 to elaborate on the difference and similarities between services. Data were entered into SPSS (IBM, Version 20) and the data was analysed using appropriate methods taking into account parametric assumptions.

Ethical approval for this study was obtained from the NRES Committee North West Preston (11/NW/0503) and all parents/guardians gave informed written consent before taking part for themselves and for their child. If children were over 11 years old they were also asked for their permission to consent, in addition to their parents.

## Results

### Hospital descriptions

In assessing DGA services it is important to note both the differences and similarities between the ways the hospitals are organised and the way care is provided. All hospitals were attended for a period of two months when clinics were operating as usual i.e. no long-term staff sickness or estate issues. Information about these hospitals is taken from the time the researcher attended and may have changed since (Additional file 1: Table S1).

It should be noted that during the two month research period theatre sessions were cancelled at hospitals for various reasons (illness, no anaesthetist available, etc.) therefore patient sessions may not add up to the total anticipated if every session had occurred as planned. Due to the high consent rate (93%) out of those who attended the clinic days there is a high degree of confidence in the relative values shown despite not all patients being included. When including those who failed to attend (who could not be approached as they did not receive treatment on that day) the consent rate is 75%. All tables in the results section are based on the number of children seen and consented at the hospital site (n).

#### Assumptions

Before analysis was completed assumptions required for parametric tests were explored. Index of multiple deprivation failed assumptions of normality (Kurtosis 2.977 p <0.05) and homogeneity of variance (F(5,430) = 2.654 p = 0.022). Therefore the variable was transformed using natural log F (5,430) = 1.829, p = 1.06.

Data for Referral to Treatment (RTT) wait time (in days), number of teeth extracted and age were also skewed and homogeneity of variance assumption not met. However, transforming these did not have an effect on normality and homogeneity of variance and therefore all further analysis on all other variables utilised non-parametric tests.

#### Analysis

Descriptive statistics (Additional file 2: Table S2) indicated there were few children who were born in another country and there was no statistically significant difference between those attending different hospitals. However, there did seem to be a great level of relocation for those attending Hospital 1 with almost a fifth of the children having moved into this area after they were born. Additionally there was a difference in main/first language spoken by attendees with Hospital 1 having a fifth of attendees stating English was not their main or first language and 7% requiring a translator at the hospital (Additional file 2: Table S2).

Treatment provided throughout the six hospitals varied (Additional file 2: Table S2). The majority of patients received extraction of deciduous teeth. However two hospitals also provided some form of restoration under General Anaesthetic (either alone or with additional extractions). Only one of these hospitals carried this out on sizable proportion of patients. Hospital 6 treated just over a quarter of patients (26%) using some form of restoration to the tooth. No other hospital noted they performed any restorative work under General Anaesthetic, only extraction of teeth. Reasons for this are explored further in the discussion.

To explore any difference between the hospitals and the number of teeth extracted, Kruskal Wallis was performed given the non-normal distribution and violation of homogeneity of variance. A significant difference was detected 88.588 (5) p= 0.0001 therefore the null hypothesis was rejected and pairwise multiple comparisons computed. The pairwise comparisons (Table 1) generated through SPSS compare pairs of groups based on rankings created using data from all groups, as opposed to just the two groups being compared, these tests are known as Dunn-Bonferroni tests [16]. Hospital 1 had on average a higher number of extractions compared to other hospitals in the study, which ranged up to a full clearance with all 20 teeth being removed (Additional file 2: Table S2).

**Table 1:** **Dunn-Bonferroni tests - pairwise comparisons between hospital and number of teeth extracted**

	**(I) Hospital**	**(J) Hospital**	**Test statistics**	**Std. error**	**Sig**	**Adjusted sig.**
Pairwise multiple comparison	1	2	19.381	21.794	0.374	1.000
		3	152.315	24.490	0.000	0.0001
		4	132.506	18.816	0.000	0.0001
		5	82.891	21.764	0.000	0.002
		6	112.418	16.535	0.000	0.0001
	2	3	132.935	28.226	0.000	0.0001
		4	113.125	23.473	0.000	0.0001
		5	63.510	25.922	0.014	0.214
		6	93.038	21.688	0.000	0.0001
	3	4	−19.810	25.996	0.446	1.000
		5	−69.424	28.226	0.014	0.209
		6	−93.897	24.396	0.102	1.000
	4	5	−49.615	23.473	0.035	0.518
		6	−20.088	18.693	0.283	1.000
	5	6	29.527	21.688	0.173	1.000

Examining the rates of previous DGAs for the participating child or any other children within their household was remarkably high ranging from 33% to 59% throughout the hospitals (Additional file 2: Table S2).

Further variables were explored to determine if there were differences between those attending the six hospitals in (data were gained from self reported parent questionnaires): regular dental attendance (child attending at least once a year), relevant medical issues or preventative treatment discussed between the parent and dentist in order to potentially account for the differences seen in repeat DGAs and number of teeth needed to be extracted (Additional file 2: Table S2). Hospital 1 had a higher percentage of irregular dental attenders, while Hospital 6 had a higher percentage of children with reported medical issues (it should be noted preventative treatment, such as fissure sealants, may have only been discussed with parents and not necessarily applied by the dentist or other health professional, additionally due to recall bias parents in some circumstances may have forgotten that these preventions have been used).

**Figure 1:**
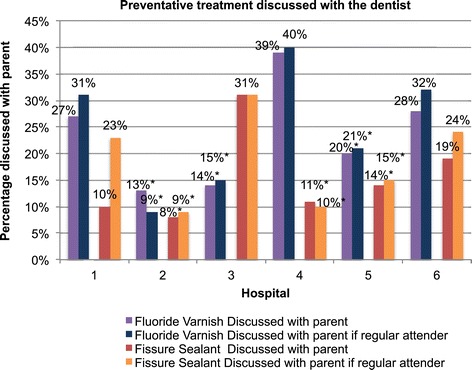
Percentage of GDP preventative measures discussed across Hospitals. *Individual difference detected for hospital 2 and 5 comparing against the collective group, which were significant for both preventative treatments and for hospital 3 for fluoride varnish and hospital 4 for fissure sealant.

Attendees within certain geographic locations had fewer GDP related preventative measures discussed (as noted from parent self complete questionnaires) (Figure 1). Evidence of a difference was detected between regions (defined by hospital attendance) in relation to; fissure sealant discussed with parents (any) x^2^ = 20.255 (5), p = 0.025, Fissure sealant discussed with regular attending parents x^2^ = 13.090 (5), p = 0.023 and fluoride varnish discussed with parents (any) x^2^ = 15.895 (5), p = 0.007 (corrected for multiple comparison p <0.025).

Failure to attend was an issue at all hospitals, with three having almost a fifth of children failing to attend on the day of their operation (Additional file 1: Table S1) no significant difference was observed between hospitals x^2^ = 6.398 (5), p = 0.269.

A one-way ANOVA was conducted (Table 2) on the transformed IMD score (natural log transformation). There was a significant difference detected between hospitals for the IMD given for each participant F (5,421) = 6.272, p = 0.0001. To determine where possible difference between each hospital lay, a Games Howell post hoc procedure was performed (Table 3). This was chosen over Bonferroni as the sample sizes between hospitals were different and this provides the best performance on such data [17]. Significant differences were observed between a number of hospitals. Hospital 2 and 3 had, on average, lower deprivation scores, with the remainder scoring similarly higher deprivation scores. While this indicates the hospitals, included in the study, covered varying regions with a variety of deprivation backgrounds based on IMD, these data should be linked back to the population of the surrounding area, i.e. if the average deprivation score of those attending for GA extraction were significantly different to the overall population of each hospital region. This was calculated using the same IMD generator but using the whole population postcode data. Analysis indicated Hospital 3 and 4 had a significantly higher IMD than the general population of the region served by the hospital, following correction for multiple comparisons (Table 4). However for all hospitals, patients who attend had on average higher IMD scores (and hence higher levels of deprivation) than the general population of that area with the majority showing moderate evidence of a difference. No significant difference was observed between hospitals and age at operation using Kruskal-Wallis H(5) 7.614, p = 0.179.

**Table 2:** **Summary statistics - transformed (natural log) index of multiple deprivation by hospitals attended and age by hospital attended**

	**IMD transform**	**95% CI**		**Age**		
**Hospital**	**Mean (s.d.)**	**Lower bound**	**Upper bound**	**Median**	**Min**	**Max**
1 (115)	1.628(.239)	1.5840	1.6724	5.917	1.50	13.17
2 (46)	1.537(.227)	1.4698	1.6045	6.583	2.17	13.83
3 (30)	1.401(.285)	1.2942	1.5068	6.667	3.83	13.92
4 (73)	1.656(.200)	1.6096	1.7027	6.667	1.83	13.42
5 (43)	1.644(.239)	1.5705	1.7181	7.000	3.67	12.42
6 (120)	1.560(.251)	1.5516	1.6422	6.333	1.75	16.42
Total	1.600(.246)	1.5766	1.6234	6.417	1.50	16.42

**Table 3:** **ANOVA multiple comparison of hospital by IMD - Games Howell correction**

					**95% CI**	
**(I) Hospital**	**(J) Hospital**	**Mean difference**	**Std. error**	**Sig.**	**Lower bound**	**Upper bound**
1	2.00	.09102	.04019	.220	-.0261	.2081
	3.00	.22773^*^	.05655	.003	.0586	.3969
	4.00	-.02798	.03230	.954	-.1211	.0651
	5.00	-.01608	.04283	.999	-.1413	.1092
	6.00	.03133	.03195	.924	-.0605	.1231
2	3.00	.13671	.06179	.250	-.0461	.3195
	4.00	-.11901^*^	.04078	.050	-.2379	-.0001
	5.00	-.10710	.04955	.267	-.2516	.0373
	6.00	-.05970	.04051	.682	-.1777	.0583
3	4.00	-.25571^*^	.05698	.001	-.4260	-.0855
	5.00	-.24381^*^	.06355	.004	-.4314	-.0562
	6.00	-.19640^*^	.05678	.015	-.3661	-.0267
4	5.00	.01190	.04340	1.000	-.1150	.1388
	6.00	.05931	.03269	.459	-.0349	.1535
5	6.00	.04741	.04314	.880	-.0787	.1735

*Significantly different following Games Howell.

**Table 4:** **One sample T test for IMD - study (study) mean and population (pop) mean**

	**Study sample IMD**			**95% CI**		**Pop IMD**	**One sample T test**
**Area**	**N**	**Mean**	**s.d.**	**Lower**	**Upper**	**Mean**	
1	115	45.776	19.330	42.205	49.347	43.56	t(114) = 2.578, p = 0.011, dif 4.46 95%CI 1.075-8.217
2	46	36.831	17.680	31.581	42.082	31.08	t(45) = 2.645, p = 0.011, dif 6.880 95%CI 1.645-12.116
3	30	28.129	16.648	21.912	34.346	17.05	t(29) = 3.645, p = 0.001, dif 11.079 95%CI 4.862-17.296*
4	73	47.696	19.388	43.173	52.220	41.01	t(73) = 3.117, p = 0.003, dif 7.091 95%CI 2.558-11.624*
(5 & 6)	163	44.647	21.385	41.339	47.954	43.45	t(162) = 0.714, p = 0.476, dif 1.19663 95%CI -2.110-4.504

*Significantly different following Bonferroni correction p < 0.008. Area 5 and 6 combined as they served the same population.

A t test was carried out given the only available data for area IMD was the mean IMD score meaning a t-test was the most appropriate statistical test to use. Area IMD score was based on information gained from PCO/PCT data from Public Health England, 2010 information (http://www.apho.org.uk/resource/item.aspx?RID=110540).

## Discussion

This study aimed to explore services where children were scheduled to receive dental extractions under GA. This was achieved by randomly selecting 6 hospitals throughout the North West England. In addition, the pre-assessment procedure and use of services before children were referred for this operation were considered using information from both a questionnaire to parents and access to referral and consultation notes. This enabled a deeper understanding of the various influences and determinants that may result in the need for a DGA from a service perspective (this issue is being explored further from both a patient and dental perspective in a companion paper [18]). Additionally, with the disbanded PCTs merging over wider geographic regions (NHS England Area Teams) it was thought that an opportunity to establish commissioned services that reflected best practice and enabled referring practitioners to understand the offer more clearly than at present.

In order to address the differences observed between hospitals the variables described in the results section were clustered into themes. Each one of these will be addressed throughout the discussion in order to establish the differences and similarities between service design and organisation within the assessed hospitals.

### Estate issues

#### Hospital setting

A child or families’ experience would have been quite different in each hospital. These differences ranged from children being assigned a bed, recovering in an area with other children, to the structure of the service delivery and wait time during the day. Some hospitals (Hospital 1,3,6) saw children all together for their pre-assessment, which meant all children arrived at the same time with the last child potentially waiting for their operation for over 5 hours. Other hospitals (2,4,5) saw children one by one or in smaller cohorts, which reduced their time at the hospital. These could all have varying impacts on the family for example waiting for a considerable amount of time whilst being starved could be troubling for young children [19], additionally recovery with others could cause further anxiety and add to an already stressful situation. The reason for a collective appointment time vs. individual times was largely due to the physical layout and capacity of the hospitals. Those hospitals with small waiting areas would typically offer individual patient appointment/start times and those with larger facilities, or waiting and pre-assessment facilities at some distance from operating theatres would offer the same appointment start time to all patients.

#### Child friendly environment

The nature of the clinics was often dictated by their setting; for example clinics could be on children’s or adult wards, or be mixed with children waiting for various other procedures or be exclusively for those attending DGA. The majority of hospitals had entertainment in the form of television, games and toys, and such activities have been shown to be invaluable in creating the opportunity to support children’s psychological wellbeing and a positive hospital experience [20]. Two hospitals had play specialists throughout the time the observer was present. These individuals talked children through the procedure using props, books and toys in a manner they could understand. Research has suggested children provided with information about their care in a hospital setting consequently felt more prepared and less anxious about their operation and treatment [21].

### Population issues

It was apparent a greater proportion of children who attended Hospital 1, compared to other hospitals, had previously lived in another geographic area and had a fifth of parents whose main language was not English. Additionally patients attending Hospital 1 required significantly more teeth extracted (on average 8). There were also significant wait times with a large number of children referred into this service requiring treatment. Therefore Hospital 1 appears to be in an area, which not only experiences a significant oral health problem in children but also has additional barriers to prevention and information being distributed.

The difficulty in providing regular support and information with frequent movement and a variety of languages spoken could impact on a continuous and consistent preventative message being successful [22]. It also indicates any interventions to reduce the number of DGAs would need to take into account language barriers and increased population mobility. This would become problematic if, for example, there was an attempt to establish a Public Health measure such as the implementation of water fluoridation or a school based varnish scheme, which only serves a local or specific area where families may not reside for sufficient time to gain benefit.

### Referral and treatment issues

#### Wait time

The impact of a prolonged wait time is explored in a separate but connected paper [23], which discussed the negative effects that could be experienced during the wait for DGA, such as pain, sleepless nights, and missed school time. Wait time varied between hospitals, with, on average, an 8-month wait. These differences could be due to a number of factors, hospitals with high referrals but fewer clinics to treat children and those with various long term staffing issues resulting in reduced capacity. Such services found themselves in a vicious circle of continuous referrals into the service with little means of addressing the wait list. As such wait lists become permanent features of such services. Wait list initiative programmes may be of little value given the timing that such initiatives take place (evening and weekend), the need for theatres, complex staffing and the high rate of Fail to Attend (FTA) seen. DGA services should also be held to the national 18-week referral to treatment target. Some services fall outside of this target as they are non-Consultant led, however, there is little justification for an exception to this and the DoH clearly stated this:

*“Dental care provided under general anaesthesia in secondary care (even where the treatment is carried out by a primary care dentist) is covered by RTT. For these dental pathways, the decision to include them within the scope of RTT was taken on the basis that these patients are typically from vulnerable groups (mainly children but also some adults with learning disabilities etc.) and it would be appropriate for them to be included in RTT. The rationale is that there has to be a consultant involved in their care as by law, general anaesthesia must be carried out in a hospital setting under the care of a consultant anaesthetist. This approach has received support from dental colleagues within the NHS”* [24].

#### Prevention and previous treatment

Certain hospitals had a higher number of repeat DGAs or more than one DGA occurring within a family unit than others. Figures indicated two thirds might attend again for a further DGA or have a sibling attend for the same procedure. This suggests more needs to be done with those actually being seen/referred into the hospital to prevent such occurrences. Previous research has shown those who are referred for DGA have not always responded to simple preventative messages, therefore a more active intervention with these families may be needed [25,26]. A prevention clinic had been recently introduced at one hospital which parents had to attend before their child could have their teeth extracted, at this time it is not known if this intervention has impacted on repeat DGA or future caries experience given its recent introduction. A difference was also seen in the prevention and advice given to parents by their own dentists or other healthcare professionals before the operation, particularly for those seen at Hospital 5, potentially contributing to the need for additional DGAs with a much smaller percentage having discussed preventative advice with their GDP. It could be referral into the DGA pathway is an opportunity to attempt to encourage good oral health not only for the child being seen but also for the family, particularly given the NHS strategy of ‘Making every contact count’ [27]. Previous studies have shown that a child undergoing DGA may motivate parents to improve their child’s oral health, at least in the short term and parents indicated they would welcome a variety of health care interventions at this stage [28,29]. Without any effective advice or prevention families could be doomed to repeat negative behaviours, that will require future DGA within the family unit.

Hospital 6 was a tertiary service and also saw a greater proportion of children referred with medical or behavioural problems or who were unable to receive DGA through other facilities. This may be one of the reasons for the inflated repeat DGA rate seen at this hospital, given these issues may contribute to difficulties in both maintaining good oral health and being able to treat a patient in primary care. This is an important point; as in theory, severe caries resulting in a child attending purely for ‘outpatient DGA’ (therefore not incorporating additional medical indicators) should be a preventable situation. However this may not be the case for certain children with special needs where one of the main reasons for requiring a GA cannot be removed. It was also noted that Hospital 6 was the only hospital, which routinely both restored and extracted teeth with all other hospitals extracting teeth under GA in the majority of cases. Additionally, the sessions were mixed with some children attending for more complex procedures that were not necessarily limited to extraction (when this was the case these children were not included). This offers one reason why restorations were a part of treatment plans. However, it may have also been a factor in the number of repeat DGA’s seen, as other hospitals take a more radical treatment approach. Alternatively, it could be a factor of the referred population where restoration is more appropriate than extraction.

There are a number of limitations regarding the study which should be acknowledged and taken into account in relation to conclusions drawn. Data collected from participant questionnaires were based on parental (and at times child) self report and should be treated with caution, given responder bias. It was originally thought that parent responses could be validated against dental referral notes, however in the majority of cases an adequate dental history was not recorded. Self report data can be skewed either by what a participant remembers or if they feel they should answer a question in a certain way therefore while data gives an indication of aspects such as dental attendance and prevention advice being administered these data may be either under or over reported. In addition given each hospital was visited for a period of two months it is acknowledged that services may have changed after data collection or new protocols put in place. Despite the limitations discussed, the use of triangulation allows a number of methods to be utilised for completeness in exploring this area combining data from both participant questionnaires, dental referral and clinical notes and qualitative interviews (which are explored in Part 2 of this work).

## Conclusion

It was observed that DGA services are defined by the processes and estate issues within each hospital, rather than by formal service specifications. However, given these current constraints, there are aspects of the services that can be modified to improve patient experience. These include treatment from children’s GDPs during the wait for their DGA procedure (for example pain management), and prevention advice to reduce the likelihood of a repeat DGA. It appears that advice or preventative treatment possibilities are not discussed with parents/guardians in a consistent fashion. Discussions occur before the operation with their own GDP, in preventative clinics or during pre-assessment with those who will be operating on their children later. In addition, preventative work can be carried out at the time of the operation, for example, fissure sealing teeth. This was undertaken at Hospital 6; possibly due to the high level of children referred with various behavioural or related issues meaning this may be the only opportunity to administer this type of treatment. However, the fact fissure sealants can be delivered in this environment is something that should be considered for all DGA services. Other reports have discussed the possibility of tooth extractions performed in this way may not always allow prophylaxis at the same time [30] therefore a separate session may be needed to carry out this preventative treatment.

A ‘one size fits all’ approach may not be possible across all DGA services given: the diverse reasons for referral, numbers of patients treated and the dental treatment required, however best practice could be shared with certain qualities and processes incorporated throughout. The differences between dental services carried out in a hospital setting have been observed across various areas of England. A study exploring DGA in Yorkshire and Humber [31] indicated the variation in the assessment and organisation of DGA lists could impact on the number of DGA operations carried out [32]. Additionally, elements such as an individual’s relocation to another region, first/main language spoken and pattern of dental attendance can make the ability to address the problems that lead severe decay in young children more complicated. In certain cases the first time a child engages with a dentist and has any form of dental treatment may be via a referral for dental general anaesthetic, and this is discussed further in Part 2 [18]. Therefore it is vital to engage with both the child and family at this stage and attempt to start a pattern of dental attendance and to ensure this experience does not create persistent dental anxiety.

**Figure 2:**
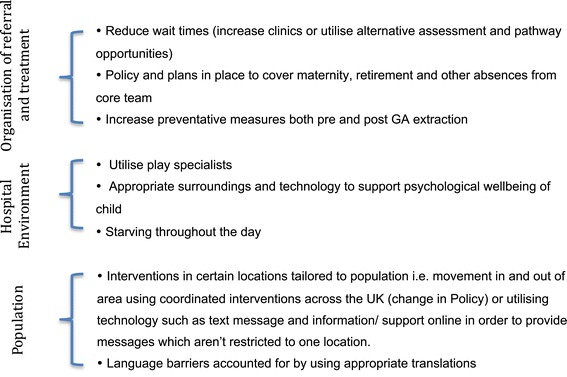
Key features that should be included within a DGA service.

Although both hospitals and patients have differing obstacles, which may require additional assistance and interventions, services across England could look to share best practice and adopt processes that have been shown to work well. DGA services should be based on informed and intelligence led commissioning practices at the heart of which should be a robust health needs assessment. Following the assessment of need, service specifications should reflect the local position with respect to the individual hospitals but key elements of the service should always be included, these are described in Figure 2.

These areas are explored further in Part 2 of this work which utilises qualitative interviews with parents and key players throughout the GA pathway to elicit a greater understanding of these factors and their impact.

This paper has demonstrated a DGA landscape of disparate services many of them reflect the influence of both the historic development of these services within hospitals and the varying requirements of the patients who attend. There is a clear need to reform these services so they are centred on patient needs, include elements of prevention and address repeat DGA attendance and delays in RTT. This work has identified elements of best practice that should be incorporated into service specifications in the future.

## Additional files

Additional file 1: Table S1. Hospital Service delivery and organisation breakdown. FT = Foundation Trust, CT2 – Core training 2 previously known as SHO, CDS = Community Dental Service. Staffing issues refer to staff leaving due to retirement, maternity or other personal reasons and a delay in implementing new staff or finding appropriate replacements. Additional file 2: Table S2 Descriptive Statistics around population attending for DGA. P = Permanent teeth, D = Deciduous Teeth, P&D Permanent and Deciduous teeth. 25%^a^/50%^b^ of cells have expected count of less than 5 therefore results should be viewed with caution. *indicates where statistical difference occurs. Medical history % out of those who had accurately reported records.

## Abbreviations

GA: General Anaesthetic
DGA: Dental General Anaesthetic
GDP: General Dental Practitioner
RTT: Referral to Treatment Time
FT: Foundation Trust
FTA: Fail to Attend
CT2: Core training 2 previously known as SHO
CDS: Community Dental Service
